# High prevalence of *Babesia microti* in small mammals in Beijing

**DOI:** 10.1186/s40249-020-00775-3

**Published:** 2020-11-11

**Authors:** Chun-Yan Wei, Xiao-Mei Wang, Zhen-Sheng Wang, Zhi-Hua Wang, Zeng-Zhi Guan, Lian-Hui Zhang, Xiang-Feng Dou, Heng Wang

**Affiliations:** 1 Department of Microbiology and Parasitology, Institute of Basic Medical Sciences, Chinese Academy of Medical Sciences & School of Basic Medicine, Peking Union Medical College, 5# Dong Dan San Tiao, Dongcheng District, Beijing, 100005 China; 2 Institute for Infectious Disease and Endemic Disease Control, Beijing Center for Diseases Prevention and Control (Beijing Center for Preventive Medical Research), No. 16 Hepingli Middle Street, Dongcheng District, Beijing, 100013 China; 3 Department of Immunology, Guilin Medical University, Guilin, 541000 Guangxi China

**Keywords:** *Babesia microti*, Genotype, Molecular epidemiology, Small mammal, Beijing, Tick-borne infectious disease, Risk factor

## Abstract

**Background:**

Babesiosis is an emerging tick-borne zoonotic infectious disease. *Babesia microti* is responsible for most cases of human babesiosis globally. It is important to investigate the prevalence of *B. microti* in the mammalian host population of a specific region in order to elucidate mechanisms of pathogen transmission and to define geographic areas where humans face the greatest risk of exposure. The aim of this study is to understand the prevalence and genotypes of *B. microti* in the small mammals that are found in Beijing, China.

**Methods:**

We trapped small mammals from all of the 16 urban, suburban, and outer suburban districts of Beijing during the years 2014, 2017 and 2018. Genomic DNA was extracted from the heart tissues individually and the *Babesia* 18S rRNA gene was detected by PCR. The genotypes of *B. microti* were identified based on sequence alignments and phylogenetic analysis. The morphology of the parasites was observed under light microscopy. The risk factors were analyzed statistically based on both univariate analyses and multivariate logistic regression.

**Results:**

A total of 1391 small mammals were collected. Positive infection of *B. microti* was detected in 12.1% (168/1391) of small mammals from 15 out of the 16 districts. Both Kobe-type and U.S.-type *B. microti*, accounting for 9.5% and 2.7%, respectively, were identified. Classic diverse morphologic forms of *B. microti* were observed. Specific types of ecological habitats including shrub areas, broad-leaved forest, and cropland were revealed to be risk factors associated with *B. microti* infection.

**Conclusions:**

This study demonstrated the wide prevalence of *B. microti* infection in eight species of small mammals in Beijing, with Kobe-type more prevalent than U.S.-type. This study provides fundamental information for the development of informed prevention and control measures by public health authorities; the data gathered indicates a need for further monitoring of both clinical diseases in individuals presenting with babesiosis-like symptoms, as well as the infection status of ticks in high risk areas.

## Background

Human babesiosis is a worldwide emerging tick-borne zoonotic infectious disease [1–3]. It has become a public health concern with increasing numbers of human cases reported in all inhabited continents [4]. The causative agents are obligate intraerythrocytic protozoa of the genus *Babesia* [5, 6]. Among the known *Babesia* species infecting humans, *Babesia microti* is the most prevalent species [7], with cases reported from the Americas, Europe, Asia, and Australia [8]. *Babesia microti* is maintained in nature through an enzootic cycle that involves hard ticks of the family Ixodidae as the definitive host and a vertebrate as an intermediate host [6]. The primary reservoir hosts for *B. microti* are small rodents such as mice and voles [9, 10]. Investigating the prevalence of *B. microti* in small mammalian hosts in a specific region is important in order to elucidate the mechanisms by which the pathogen spreads and to define geographic areas where humans are at risk of exposure.

Beijing, as the capital city of China, is the center for international exchange, tourism, and other activities, comprising a total of 16 districts, with two urban, ten suburban and four outer suburban districts. Beijing is surrounded by mountains on three sides and has a semi-humid, seasonally temperature climate that is influenced by monsoon winds. Different geographical landscapes and vegetation distributions of Beijing make it a good natural environment for vectors and wild animals with pathogen reservoirs. *Babesia microti* infection in rodents from Beijing has been documented since 2013 [11, 12], but the whole picture of epidemiology for *Babesia* infection in small mammalian hosts in all 16 districts of Beijing were still largely unknown. The aim of this study is to understand the prevalence and genotypes of *B. microti* in the small mammals that are found in all 16 districts of Beijing.

## Methods

### Study areas, samples collection, and DNA extraction

Small mammals were captured by animal snap traps from all 16 districts of Beijing during the years 2014, 2017 and 2018. The sample sites represent five different ecological habitat types: (1) residential areas, (2) cropland, (3) shrub, (4) broad-leaved forest and (5) mixed coniferous and broad-leaved forest (mixed forest for short). The altitudes of sampling sites range from 20 to 1100 m. Approximately 140–150 traps were placed every night at every sampling location for three consecutive nights. The species of trapped small mammals were identified based on their morphology referring to Chinese monographs [13, 14]. The gender, developmental stage, and environment of the captured mammals were recorded. After that the heart tissues were isolated individually and stored in liquid nitrogen until DNA extraction. Total genomic DNA of heart tissues was extracted using the Magnetic Beads Animal Tissue Genomic DNA Extraction Kit (Catalog number: NMG601, NanoMagBio Technology, China) according to the manufacturer's instructions. Briefly, about 5 mg of heart tissue was isolated from each sample, and lysed at 68 ℃. The supernatant was then transferred to NanoMagBio S-96 automatic nucleic acid extraction platform with appropriate parameter settings. Finally, the genomic DNA was eluted in 50 μL elution buffer and stored at − 20 ℃.

### PCR detection and phylogenetic analysis

PCR targeting a specific fragment of *Babesia* 18S rRNA gene was performed using the primer pair BJ1/BN2 [15], which amplified fragments ranging 516–521 bp in length among different strains of *B. microti* according to sequences of the 18S rRNA genes of *B. microti* published in GenBank. Each amplification was carried out in a volume of 25 μl containing 12.5 μl of 2 × Phanta Max Master Mix (Catalog number: P515-01, Vazyme Biotech Co., Ltd, Nanjing, China), 1 μl of genomic DNA template, 0.75 μl of primers BJ1 and BN2 with each of 10 μmol/L. The reaction mixtures were subjected to an initial denaturation step of 3 min at 95 ºC, followed by 38 cycles of denaturation at 95 ºC for 15 s, annealing at 57.5 ºC for 30 s, and elongation at 72 ºC for 30 s. Amplification was completed by a further step of 7 min at 72 ºC. Genomic DNA isolated from *B. microti* strain Peabody mjr (Catalog number: PRA-99™, ATCC) was used as the positive control and distilled water as the negative control. Amplicons were sequenced directly in both directions with the ABI 3730 xl DNA sequencing platform. Sequence alignments and analysis were carried out using DNAMAN version 7.0 (Lynnon Biosoft, San Ramon, USA). A search for homologous sequences was performed using the Basic Local Alignment Search Tool (BLAST) from the National Center for Biotechnology Information (NCBI).

For *B. microti* PCR-positive samples amplified using primer BJ1/BN2, two overlapping fragments, with both longer than 1700 bp in length, of the 18S rRNA genes were amplified by PCR amplifications. The near full-length 18S rRNA gene sequences were obtained by assembly of the two large overlapping fragments. The primer pairs for the two amplifications were Piro1F/rRNA3′ and Piro2F/Piro6R [16, 17], respectively. The primer Prio2F was almost the same as Prio0F from reference [17] except the replacement of a “C” with “S”. The conditions for amplifying the two large fragments were the same as those mentioned above except for the extension time in 38 cycles, which was 1 min 20 s. Amplicons were also sequenced directly but using two more primers (Ba-SL and Ba-SR) besides the amplifying primers for full sequencing. Sequence assembly was performed using ContigExpress of Vector NTI Suite version 9.0 (Thermo Fisher Scientific Inc., Waltham, USA). All sequences of primers used in this study are listed in Additional file 1: Table S1; the diagram for illustrating the positions and length of primers, the amplified fragments and the near full-length 18S rRNA gene is shown in Additional file 2.

For phylogenetic analysis, nucleotide sequences of 18S rRNA genes from another 34 isolates of *B. microti* from the USA, Europe, and Asia were included for comparison. Additionally incorporated were *Babesia duncani* WA1 from the USA, *Babesia divergens* from Europe, *Babesia crassa-*like pathogen (*Babesia* sp. strain H110), *Babesia venatorum*, and *Babesia* sp. XXB/Hangzhou from China, which were included as the outgroup. All sequences used in phylogenetic analysis were downloaded from GenBank and listed in Additional file 1: Table S2. The phylogenetic tree was constructed by the Neighbor-Joining method with the p-distance model as implemented in MEGA7 [18]. Phylogenetic analysis using maximum parsimony method was also conducted to examine the reliability of the resulting phylogenetic tree.

### Morphology observation of *Babesia microti* parasites

Blood smears were made from captured animals from the district of Miyun in 2018. The smears were fixed with 100% methanol, stained with diluted Giemsa solution (Catalog number: SLBS0364, Sigma-Aldrich) for 10 min; morphology observation was performed under a light microscope at × 1000 magnification with oil immersion.

### Statistical analysis

The association between sampling time, altitude of sampling sites, ecological habitats, developmental stages, genders of collected mammals, and *Babesia microti* infection were analyzed using univariate analyses based on chi-square (*χ*^2^) test. The risk factors for *B. microti* infection were analyzed based on multivariate logistic regression. Significance level for all tests was set at *P* < 0.05. All analyses were conducted using SPSS version 19.0 (IBM Corp, Armonk, USA).

## Results

### Prevalence of *Babesia microti* in small mammals in Beijing

**Fig. 1:**
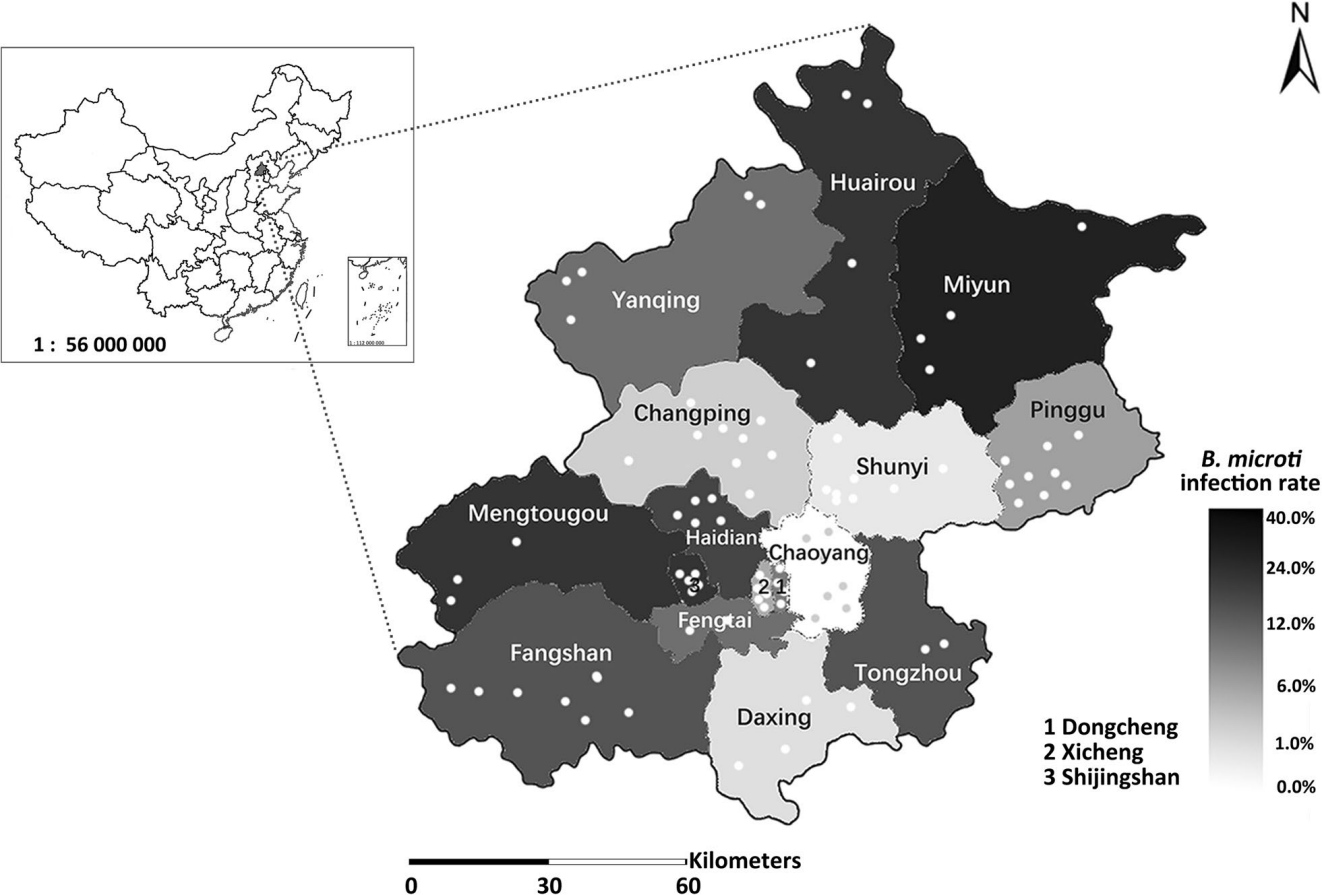
Map of *Babesia microti* prevalence in small mammals in Beijing. As is shown, *B. microti* is highly prevalent in small mammals in Beijing, with positive *B. microti* infection samples from 15 out of the 16 districts. The geographic location for each trapping locality is labeled with white or gray dots for easy recognition in the map. The prevalence of *B. microti* infection in each district is represented by different colors as illustrated in the legend at the lower right corner of the figure

A total of 1391 small mammals were trapped at 88 various sites from all of the 16 urban, suburban, and outer suburban districts of Beijing (Fig. 1, Table 1). The small mammals belong to the two orders—Rodentia and Insectivora—and represent four families, eight genera, and nine species (Table 2). Sequence alignment showed that an average of 12.1% (168/1391) small mammals were positive for *B. microti* infection (Tables 1 and 2).

**Table 1:** Prevalence of *Babesia microti* in small mammals from different districts

Districts	Regional type	No. of mammals tested	No. of positive	Positive rate (%)	Odd ratio	No. of Kobe-type (%)	No. of U.S.-type (%)
Miyun	Outer suburban	93	34*	36.6	56.47	31 (33.3)	4 (5.4)
Huairou	Outer suburban	83	18	21.7	27.14	17 (20.5)	1 (1.2)
Mentougou	Suburban	80	16	20.0	24.50	14 (17.5)	2 (2.5)
Shijingshan	Suburban	67	13	19.4	23.59	13 (19.4)	0 (0.0)
Haidian	Suburban	67	11	16.4	19.25	8 (11.9)	3 (4.5)
Fangshan	Suburban	105	15	14.3	16.33	14 (13.3)	1 (1.0)
Tongzhou	Suburban	78	11	14.1	16.09	10 (12.8)	1 (1.3)
Fengtai	Suburban	59	6	10.2	11.09	2 (3.4)	4 (6.8)
Yanqing	Outer suburban	120	12	10.0	10.89	5 (4.2)	7 (5.8)
Dongcheng	Urban	105	10	9.5	10.32	3 (2.9)	7 (6.7)
Pinggu	Outer suburban	121	8	6.6	6.94	6 (5.0)	2 (1.7)
Changping	Suburban	141	9	6.4	6.68	6 (4.3)	3 (2.1)
Xicheng	Urban	66	3	4.6	4.67	2 (3.0)	1 (1.5)
Daxing	Suburban	59	1	1.7	1.69	0 (0.0)	1 (1.7)
Shunyi	Suburban	99	1	1.0	1.00	1 (1.0)	0 (0.0)
Chaoyang	Suburban	48	0	0.0		0 (0.0)	0 (0.0)
Total		1391	168	12.1		132 (9.5)	37 (2.7)

*Among the 34 samples of Miyun, there was one sample infected by both Kobe-type and U.S.-type *B. microti*

**Table 2:** Prevalence of *Babesia microti* in small mammals of different species

Order	Families	Subfamily	Genus	Species	No. of tested	No. of positive	Positive rate (%)	No. of Kobe- type (%)	No. of U.S.- type (%)
Rodentia	Cricetidae	Cricetinaeh	*Tscherskia*	*Tscherskia triton*	12	4	33.3	3 (25.0)	1 (8.3)
		Arvicolinae	*Myodes*	*Myodes rufocanus*	4	1	25.0	1 (25.0)	0 (0.0)
	Muridae	Murinae	*Niviventer*	*Niviventer confucianus*	188	58*	30.9	51 (27.1)	8 (4.3)
			*Apodemus*	*Apodemus speciosus*	120	19	15.8	14 (11.7)	5 (4.2)
				*Apodemus agrarius*	82	11	13.4	10 (12.2)	1 (1.2)
			*Mus*	*Mus musculus*	197	22	11.2	13 (6.6)	9 (4.6)
			*Rattus*	*Rattus norvegicus*	776	52	6.7	39 (5.0)	13 (1.7)
	Sciuridae	Xerinae	*Sciurotamias*	*Sciurotamias davidianus*	7	0	0.0	0 (0.0)	0 (0.0)
Insectivora	Soricidae	Soricinae	*Sorex*	*Sorex unguiculatus*	5	1	20.0	1 (20.0)	0 (0.0)

*Among the 58 positive rodents of *Niviventer confucianus*, there was one infected by both Kobe-type and U.S.-type *B. microti*

Among the nine species of trapped small mammals, the brown rat (*Rattus norvegicus*) accounted for the largest proportion of any species tested, comprising 55.8% (*n* = 776), whereas *Myodes rufocanus* made up the smallest proportion, representing only 0.3% (*n* = 4). With the exception of the rock squirrels (*Sciurotamias davidianus*), eight of the nine species were found to be positive for *B. microti* infection. The *B. microti* positive infection rates ranged from 6.7% (52/776) of *Rattus norvegicus* to 33.3% (4/12) of the greater long-tailed hamster (*Tscherskia triton*) (Table 2).

Positive infected small mammals were found from all of the urban, suburban, and outer suburban districts except for the suburban district of Chaoyang (Fig. 1, Table 1). Rodents from the outer suburban district Miyun exhibited the highest *B. microti* infection rate of 36.6% (34/93), and the infection rates of small mammals from districts of Miyun, Huairou, Mentougou, Shijingshan, Haidian, Fangshan and Tongzhou were significantly higher than that in Shunyi when it was used as a control (odds ratios: 56.47, 27.14, 24.50, 23.59, 19.25, 16.33, 16.09, respectively; *P* = 0.000 for all).

**Fig. 2:**
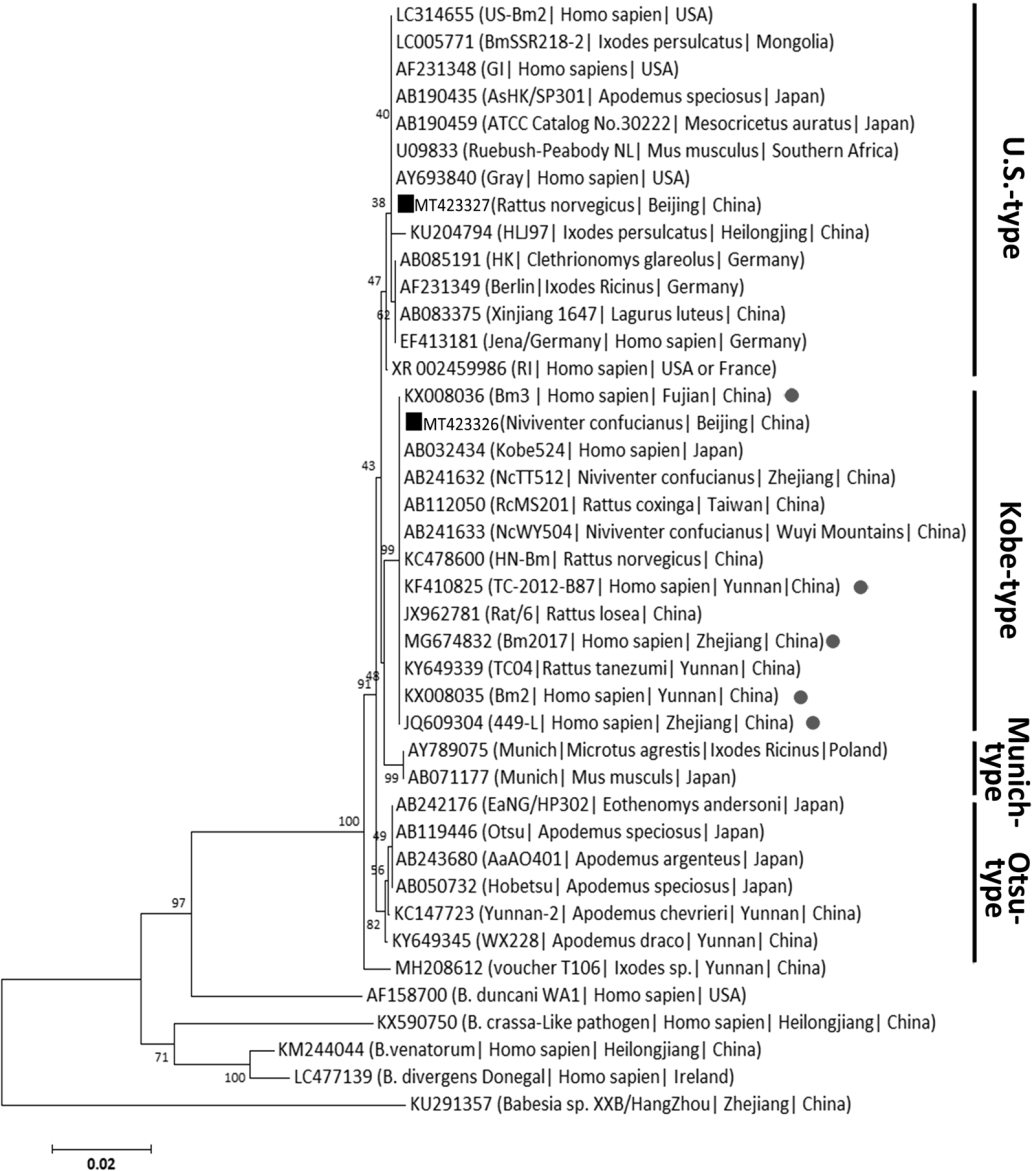
Neighbor-joining phylogenetic tree based on the comparison of *Babesia microti* 18S rRNA gene sequences obtained in this study with *B. microti* reference strains. *Babesia duncani* WA1*, Babesia crassa-*like pathogen (*Babesia* sp. strain H110), *Babesia venatorum*, *Babesia divergens* and *Babesia* sp. XXB/Hangzhou were used as the outgroup. The number on each branch denotes the percent occurrence in 1 000 bootstrap replicates. Black squares stand for sequences identified in this study. Gray dots indicate human *B. microti* babesiosis in China. Branch lengths are drawn proportional to the estimated sequence divergence. Phylogenetic tree based on maximum parsimony method was also conducted to examine the effect of the resulting phylogenetic tree (data not shown)

Near full-length 18S rRNA gene sequences were recovered from 59 out of 168 *B. microti* positive samples in this study, comprising two unique sequences. The two sequences were deposited in GenBank with the accession numbers MT423326 and MT423327, respectively. There are 58 sequences, 1752 bp in length for each one, identical to MT423326, and one sequence, 1747 bp in length, identical to MT423327. Phylogenetic analysis revealed that MT423326 and MT423327 belonged to Kobe-type and U.S.-type *B. microti*, respectively (Fig. 2).

**Fig. 3:**
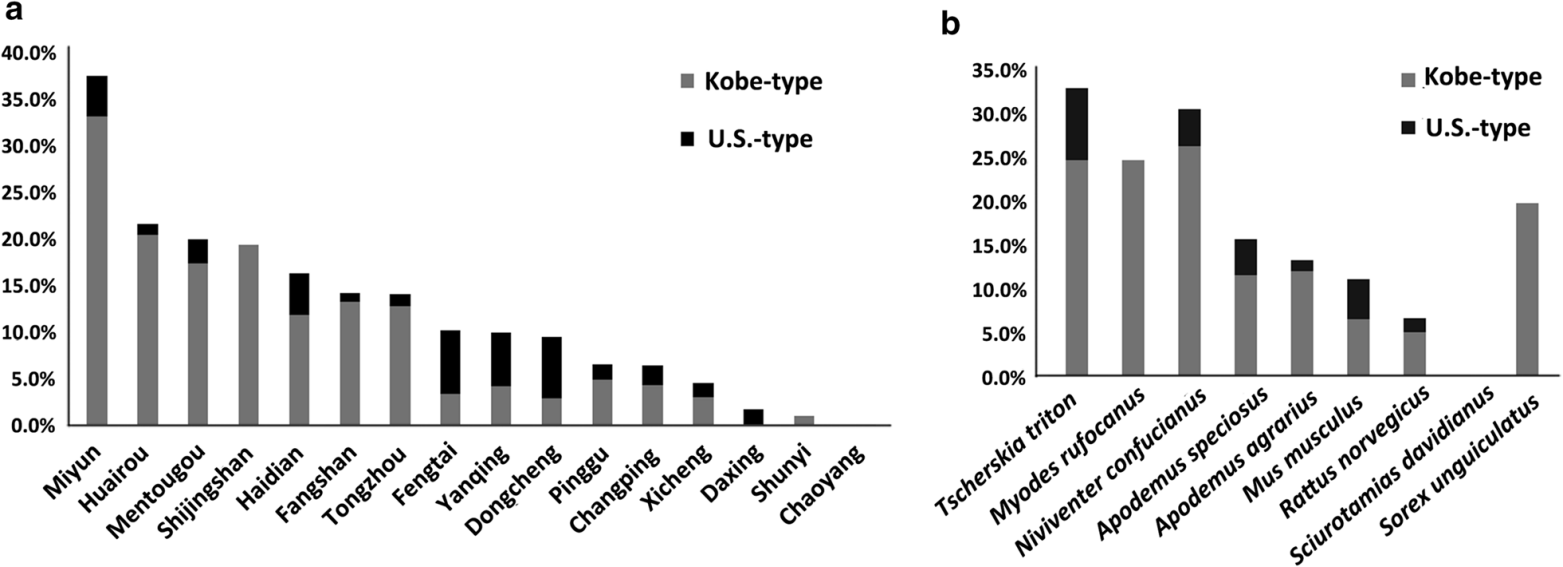
Prevalence of both Kobe-type and U.S.-type *Babesia microti* from different districts (**a**) and in different reservoir hosts (**b**). The y axis represents the positive infection rate of *B. microti* in both (**a**) and (**b**), while the x axes in (**a**) and (**b**) represent names of different districts and different species of small mammals, respectively. As is shown, Kobe-type *B. microti* is more prevalent than U.S.-type in nine out of the 15 positive districts (**a**) and in all of the eight species of small mammalian hosts (**b**)

In this study, Kobe-type *B. microti* was found to be the dominant population in Beijing (Fig. 3), as it was revealed to be more prevalent than U.S.-type in nine out of the 15 positive districts. The prevalence of the two types in the other five districts, including Dongcheng, Xicheng, Fengtai, Changping, and Yanqing (Fig. 3a, Table 1), was similar. The only positive sample detected in the suburban district of Daxing was the U.S.-type, which is the first demonstration of an existence of U.S.-type *B. microti* in this area. Kobe-type *B. microti* was also more prevalent than U.S.-type in all of the positive species, except for the house mouse (*Mus musculus*), for which the prevalence of the two types of *B. microti* was similar (Fig. 3b, Table 2). Furthermore, one rodent of *Niviventer confucianus* from the Miyun district was positive for both of the two genotypes of *B. microti*, demonstrating co-infection of different genotypes in some of the reservoir hosts.

### Diverse morphologies of *Babesia microti*

**Fig. 4:**
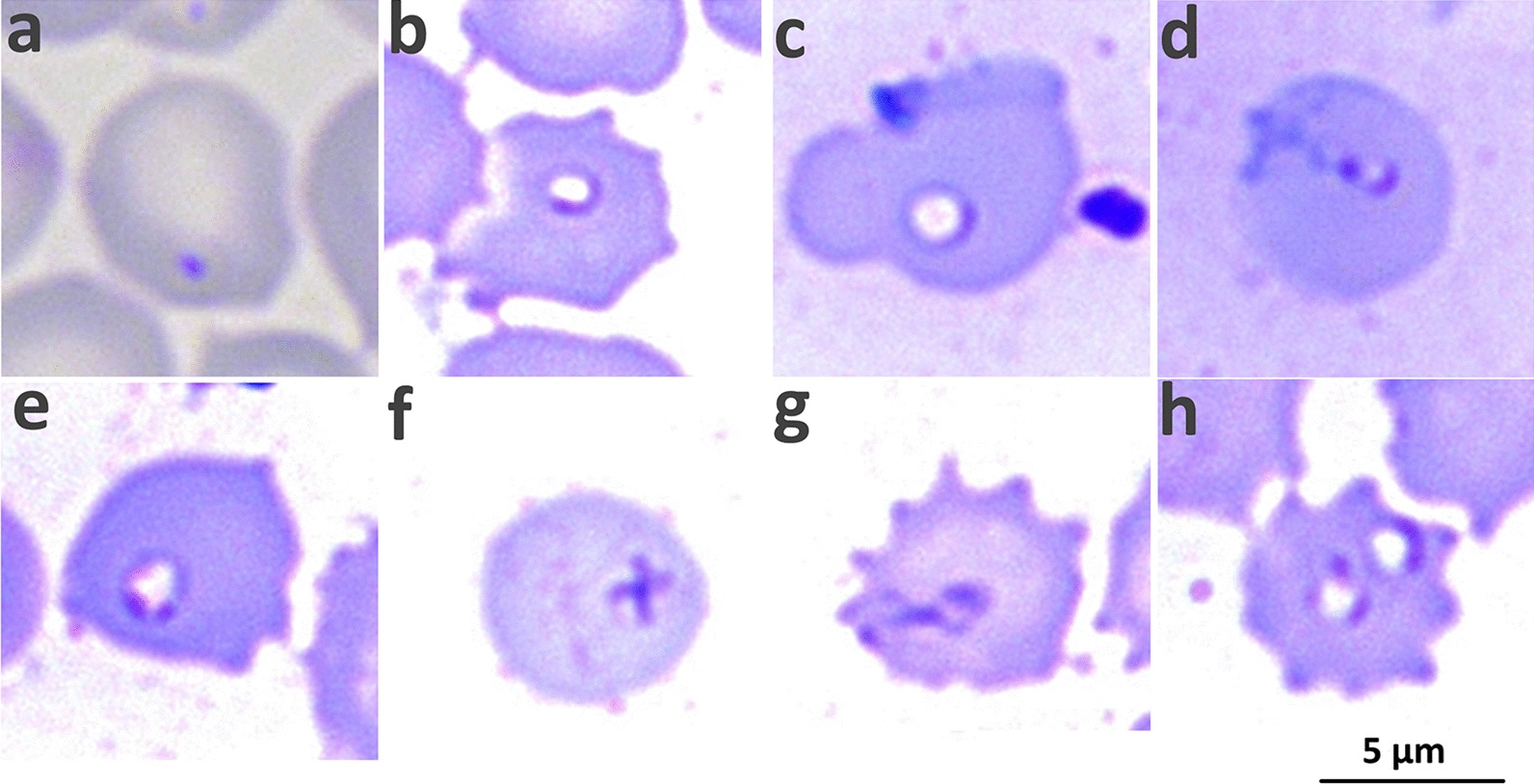
Photomicrographs of Giemsa-stained thin smear blood films. As is shown, classic diverse forms of *Babesia microti* parasites in rodents’ erythrocytes were observed, including dot form (**a**), ovoid form (**b**), ring form (**c**), pyriform (**d**), headphone form with two chromatin dots (**e**), Maltese cross form (also known as tetrad form) (**f**), and ameboid forms (**g**). Most of the infected red blood cell had one parasite (**a**–**g**), but two parasites in one red blood cell were also observed (**h**)

Of the 33 samples from Miyun in 2018, 27.3% (9/33) were confirmed to be positive for *B. microti* in blood smears, while 39.4% (13/33) were confirmed to be positive by PCR detection, reflecting the proven higher sensitivity of PCR than the microscopic methods in *Babesia* detection [19, 20]. Classic diverse morphologic forms of *B. microti* parasites in erythrocytes of rodents were observed from the Giemsa-stained thin blood smears (Fig. 4), including dot form, ovoid form, ring form, pyriform, headphone form with two chromatin dots, Maltese cross form (also known as tetrad form), and ameboid forms, with the first five forms more commonly observed than the other two included in this study. In particular, the Maltese cross form was occasionally observed, although it is considered as a characteristic morphological feature of *Babesia* parasites in human babesiosis. In addition, this featured form seemed to appear more frequently in U.S.-type *B. microti* than in Kobe-type *B. microti*.

### The risk factors associated with *Babesia microti* infection

The risk factors associated with *Babesia microti* infection in small mammals were analyzed from the perspectives of different sampling years, sampling altitude ranges, etiological habitats, ages, and genders of the small mammals (Table 3). Significant differences were present in all perspectives except in the sampling year. The prevalence of *B. microti* in male hosts (14.2%) was significantly higher than that in female hosts (*P* = 0.029); *B. microti* infection in adult hosts (13.3%) was significantly higher (*χ*^2^ = 8.88, *P* = 0.003) than that seen in the pubertal ones (6.2%) as depicted in Table 3. Notably, the prevalence of *B. microti* in small mammals from shrub areas, broad-leaved forests, and cropland was 27.4%, 23.0%, and 16.0%, respectively, and each was significantly higher than that found from residential areas (*P* < 0.05, Tables 3 and 4). No difference was shown between *B. microti* infection from mixed forests and that from residential areas (*P* = 0.844, Table 4). Further, the multivariate logistic regression analysis revealed that the altitude of 40–400 m, adult life stage, and ecological habitats of shrub areas, broad-leaved forest, and cropland were risk factors associated with the *B. microti* infection (Table 4). Interestingly, compared to broad-leaved forest, the mixed forest was not revealed as a risk factor, possibly because the coniferous plants might be a negative factor for the hosts to retain *B. microti* parasites, but further study needs to be done to elucidate this risk effect*.*

**Table 3:** Univariate analyses of risk factors related to *Babesia microti* infection

Variable		Sample size		*Babesia microti* infection		
		Cases	Constituent ratio (%)	Positive rate (%)	*χ*^2^	*P*-value
Year	2014	446	32.1	10.8	1.78	0.410
	2017	419	30.1	11.7		
	2018	526	37.8	13.5		
Altitude (m)	≤ 40	305	21.9	5.9	16.47	< 0.001
	40–400	903	64.9	14.5		
	> 400	183	13.2	10.4		
Gender	Female	514	39.4	9.9	4.77	0.029
	Male	790	60.6	14.2		
Age	Adult	1149	82.6	13.3	8.88	0.003
	Pubertal	242	17.4	6.2		
Ecological habitat	Residential	822	59.1	7.2	73.34	0.000
	Shrub	135	9.7	27.4		
	Mixed forest	143	10.3	8.40		
	Broad-leaved forest	191	13.7	23.00		
	Cropland	100	7.2	16.00		

This table shows that significant differences were related to different altitudes, genders, ages, and types of ecological habitat of the small mammals, but not to different sampling years, based on the univariate analyses using Chi-square test

**Table 4:** Risk factors related to *Babesia microti* infection based on multivariate logistic regression

Variable		*OR* (95% *CI*)	*P*-value
Altitude (m)	≤ 40	1	
	40–400	2.293 (1.331–3.951)	0.003
	> 400	1.071 (0.476–2.410)	0.868
Gender	Female	1	
	Male	1.370 (0.949–1.978)	0.092
Age	Pubertal	1	
	Adult	1.976 (1.114–3.504)	0.020
Ecological habitat	Residential	1	
	Shrub	4.505 (2.715–7.476)	< 0.001
	Mixed forest	1.079 (0.508–2.291)	0.844
	Broad-leaved forest	3.406 (2.057–5.638)	< 0.001
	cropland	2.122 (1.146–3.927)	0.017

This table shows that the altitude of 40–400 m, adult life stage, and ecological habitats of shrub areas, broad-leaved forest, and cropland were risk factors associated with the *B. microti* infection based on the multivariate logistic regression

*OR* odd ratio, *CI* confidence interval

## Discussion

This study was a systematical epizootic investigation of *Babesia* infection in small rodent and insectivore hosts from all of the 16 urban, suburban, and outer suburban districts of Beijing, and showed the wide prevalence of *Babesia microti* in small mammals in Beijing, with 15 districts harboring positive detection. *Babesia microti* infection failed to be detected in small mammals in Chaoyang, the only negative district, a finding possibly related to the smaller sample size in this area (Table 1). Previously, the existence of the hard ticks *Ixodes persulcatus*, *Haematopus longicornus*, and *Haemaphysalis conicinna*, which were vectors identified for *B. microti* infection in other provinces was documented [21]; these were indeed reported from several forest areas and scenic spots from six abovementioned districts, including three outer suburban districts of Miyun, Huairou, and Yanqing, and three suburban districts of Mentougou, Fangshan, and Changping [22–24]. The high prevalence of *B. microti* in small mammals from four out of the six districts in this study, strongly supports the hypothesis that there are important natural foci for human babesiosis in the four districts of Miyun, Huairou, Mentougou, and Fangshan, which demonstrated infection rates as high as 36.6%, 21.7%, 20.0% and 14.3%, respectively. These unexpected results call attention to the neglected *B. microti* transmission cycle in Beijing, and as such, it is important to closely monitor the prevalence of *B. microti* in these districts. It is worth noting that neighboring districts do not always have similar prevalence of *B. microti*. For example, infection rate in neither Pinggu nor Shunyi, the two suburban districts, was detected to be over 10.0%, although both of them were adjacent to the outer suburban district of Miyun, which presented the highest infection rate (Fig. 1 and Table 1). The similar phenomenon also occurred in Yanqing and Changping, both of which were neighboring to Huairou. These situations are probably attributable to different distribution and density of small mammals and tick vectors, as is known Miyun and Huairou have the most dense and abundant vegetations in Beijing, which might be more beneficial to the diversity and density of vectors and mammalian hosts.

Another unexpected finding was that the only two urban districts, Dongcheng and Xicheng, were also detected as positive for *B. microti* prevalence, although the infection rates were below the average, with 9.5% and 4.6%, respectively. This finding may give novel clues to the only reported human babesiosis case from Beijing: a male patient living in Xicheng diagnosed with a *Babesia* infection [25], whose infection was highly attributed to a history of labor in rural areas of the adjacent Hebei province. For this case, infection via a local tick bite should be considered. It is also important to point out that there is no positive correlation between the high infection rate and the three different district types of outer suburban, suburban, and urban ones, as the fact that the six suburban districts, including Mentougou, Shijingshan, Haidian, Fangshan, Tongzhou, and Fengtai, presented higher infection rates than the two outer suburban districts of Yanqing and Pinggu did, and Pinggu even presented lower infection rate than the urban district of Dongcheng; the other three suburban districts of Daxing, Shunyi, and Chaoyang all presented lower infection rate than the only two urban districts did (Fig. 1 and Table 1). These phenomena might be related to the relationship between small mammals and their various ecological habitats, which does strongly suggest that monitoring the prevalence of *B. microti* should not be limited to wild environments; that in areas close to residents should also be paid intensive attention to. This finding implies that tick-borne diseases, are probably closer to human habitation than people thought. Additionally, further consideration should be given for any patient exposures to certain ecological habitat types—specifically broad-leaved forest, shrub, and cropland—as this study has shown that such exposure is considered to be an important risk factor (Tables 3 and 4). Ecological habitat type of forest was also found to be an important risk factor for *B. microti* infection in other studies in Yunnan [26, 27] and in Southeast Asia [28]. Thus, these findings show that forests, the natural habitat for ticks, likely represent areas of high risk for transmission of *B. microti* to humans, and as such, people either working in or traveling to the forest, should utilize adequate protection. Based on our findings, this study not only strongly suggests that the surveillance of *B. microti* prevalence in all the 16 districts of Beijing could not be overemphasized, but also calls attention to difficult clinical infections in patients who had a history of tick bites from at-risk areas. The development of informed prevention and control measures by the public health systems is urgently needed. Further detailed molecular identification of *B. microti* in ticks and residents should be imperatively included.

Our present study revealed that both Kobe-type and U.S.-type *B. microti* were prevalent in small mammalian hosts from Beijing based on the large number of samples tested (Tables 1 and 2)—with Kobe-type more prevalent than U.S.-type. As is known, *B. microti* has been recognized as a genetically diverse complex group, generally clarified into U.S.-, Munich-, Kobe-, and Hobetsu- (or Ostu-) types [20, 28, 29]. In Beijing, only Kobe-type *B. microti*, was detected from small rodents in Miyun, and documented previously [11]. Hence, the identification of U.S.-type in this study revealed the genetic diversity of *B. microti* in small mammals in Beijing, as well as that in China as a whole. Previously, the most highly recorded genotype of *B. microti* was the Kobe-type, including those recorded from Yunnan [26, 27], Zhejiang, Fujian, and Taiwan [30, 31]. *Babesia microti* of Ostu-type was also detected from Yunnan province [26, 27]. U.S.-type *B. microti* was only reported from rodents from Xinjiang [16]. Thus, it is highly possible that U.S.-type is not the dominant population in China compared to the Kobe-type, a determination that would require further investigation with larger sample size and wider sampling areas. It is worth noting that human *B. microti* babesiosis reported in China was all clustered to Kobe-type *B. microti* (Fig. 2), implying the possible correlation between the *B. microti* infection in potential reservoir hosts and that as well in humans. It also implies the major responsible role of Kobe-type *B. microti* for human babesiosis in China, for which further focused correlational research needs to be conducted. In addition, a recent research study demonstrated that U.S.-type was more prevalent than Kobe-type *B. microti* in rodents from Southeast Asia including Thailand, Cambodia, and Lao PDR [28]. These data together show that the genotypes of *B. microti* in small mammals differ according to the different geographical regions investigated.

To our best knowledge, the greater long-tailed hamster (*Tscherskia triton*), has not been reported for *B. microti* infection before, while the other seven species involved in this study were reported in previous studies to serve as probable reservoir hosts for *B. microti* [11, 26, 31–34]. Our findings indicate the potential role of *Tscherskia triton*, with such a high infection rate of 33.3% (Table 2), for maintaining an enzootic cycle of *B. microti*, but further detailed studies are needed to confirm whether it is a reservoir host, an occasional host, or even an enhancing host. In addition, the co-infection with two different genotypes of *B. microti* in one *Niviventer confucianus* rodent was the first such co-infection identified by this study. It is possible that more co-infections will be uncovered with larger sample sizes. The co-infection prompts us to consider whether there are also dual infections occurring in humans, which would most certainly bring new challenges to the prognosis and the specific diagnosis for human babesiosis. This point deserves clinical attention, considering that little is known about the impact of co-infection on both virulence of the parasites and its possible correlation to clinical relapse.

There are some limitations of our study. (1) We did not choose nested PCR, which was used in many relevant investigations [27, 35]. However, the risks of contaminations during the performance of nested PCR are high, especially when the sample size is large (a total of 1391 samples in our study). Moreover, no significant difference in the sensitivity and that in the specificity between conventional PCR and nested PCR was reported in several other studies [36, 37]. (2) Near full-length 18S rRNA gene sequences, 1752 bp for the Kobe-type and 1747 bp for the U.S.-type in this study, were recovered from only 35.1% (59/168) of the positive samples. As is known, it is more difficult to amplify longer fragments than the shorter ones. Furthermore, genomic DNA in this study was extracted from wild small mammals, which might harbor complicated pathogens, making it more interfering in recovering targeted genes of near full-length. Molecular detection methods with higher sensitivity and specificity are worth trying in the future study.

## Conclusions

Our study revealed the wide prevalence of two genotypes of *Babesia microti* in small mammals collected from the outer suburban, suburban and urban areas of Beijing, with *B. microti* of Kobe-type more prevalent than U.S.-type. This study provides fundamental data to support the development of informed prevention and other control measures that can be enacted by the public health authorities. The data demonstrates a need for further surveillance of humans with babesiosis-like symptoms, and further surveillance of the epidemiology of *Babesia* infection of ticks in these areas of interest. Further studies are needed to fully identify the impact of *B. microti* in Beijing. These would include: (1) both molecular and serological investigations into *B. microti* infection in local residents, (2) detailed detection and genetic identification of *B. microti* in ticks, and (3) deeper understanding of the transmission scenarios from small mammals to ticks and then to highly susceptible humans.

## Supplementary information

**Additional file 1:** Sequences used in the manuscript. **Table S1.** Primers used in this study to amplify and to sequence the 18S rRNA gene sequences of *Babesia microti*. **Table S2.** All sequences used for phylogenetic analysis in this study.

**Additional file 2:** Diagram for illustrating positions and length of sequences involved in recovering the near full-length 18S rRNA gene sequences.

## Data Availability

The datasets collected and/or analyzed during the current study are available from the corresponding author upon reasonable request. Please contact author for data requests.

## Abbreviations

DNA: Deoxyribonucleic acid
rRNA: Ribosome ribonucleic acid
PCR: Polymerase chain reaction
bp: Base pair
