# Supplementary figures and images for: High prevalence of Babesia microti in small mammals in Beijing
Source: Infect Dis Poverty. 2020 Nov 11;9:155. doi: 10.1186/s40249-020-00775-3 (PMC7661193; doi:10.1186/s40249-020-00775-3)

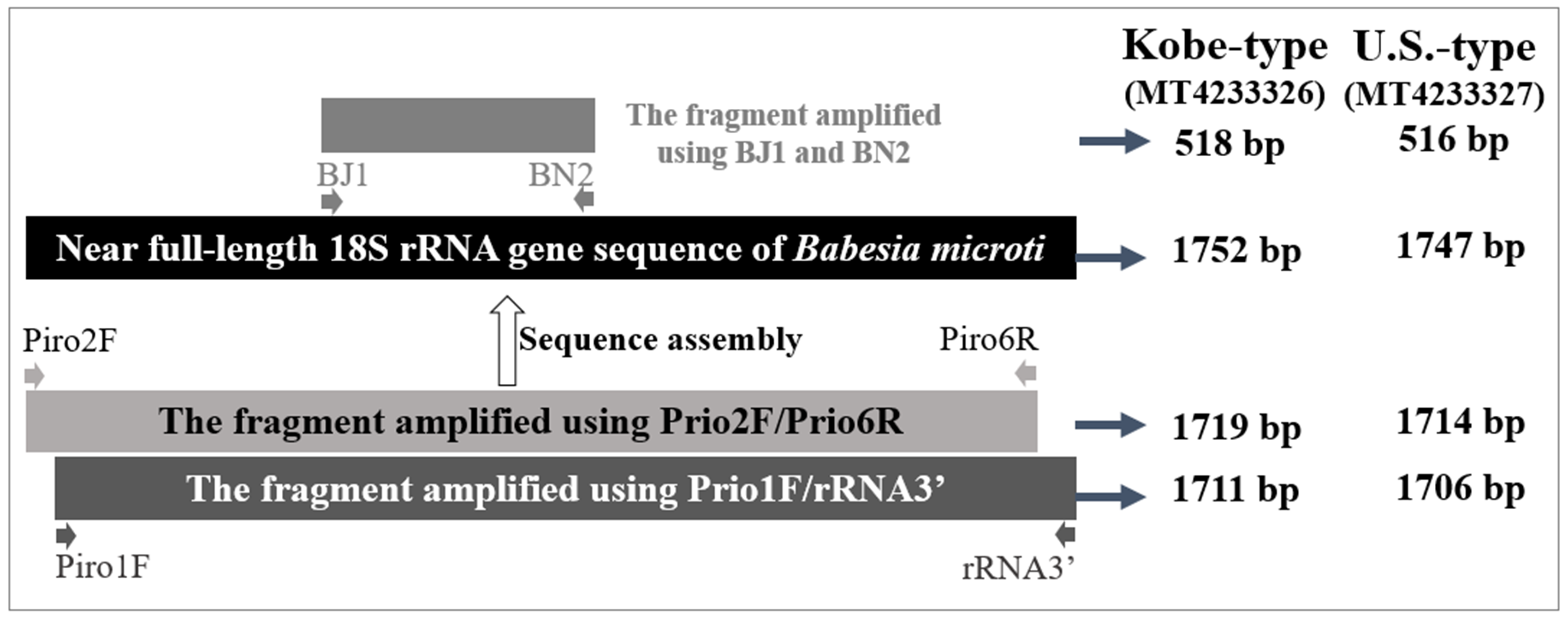

Supplement: Supplementary file 2 — Additional file 2: Diagram for illustrating positions and length of sequences involved in recovering the near full-length 18S rRNA gene sequences. [file 40249_2020_775_MOESM2_ESM.tif]
